# The Status of Asthma in the United States

**DOI:** 10.5888/pcd21.240005

**Published:** 2024-07-18

**Authors:** Cynthia A. Pate, Hatice S. Zahran

**Affiliations:** 1 Division of Environmental Health Science and Practice, National Center for Environmental Health, Centers for Disease Control and Prevention, Atlanta, Georgia

## Abstract

**Introduction:**

Asthma imposes a substantial health and economic burden on patients and their families and on the health care system. An assessment of the status of asthma in the US may lead to effective strategies to improve health and quality of life among people with asthma. The objective of our study was to assess the historical trends and current state of asthma illness and death among children and adults in the US.

**Methods:**

We assessed asthma-related emergency department visits and hospitalizations among children and adults by using data from the 2010–2021 National Health Interview Survey (NHIS), the 2010–2020 Nationwide Emergency Department Sample (NEDS), the National (Nationwide) Inpatient Sample (NIS), the Healthcare Cost and Utilization Project (HCUP), and the Agency for Healthcare Research and Quality (AHRQ). Asthma death rates were calculated by using 2010–2021 National Vital Statistics System data.

**Results:**

Asthma prevalence increased significantly among adults from 2013 through 2021 (*P *= .04 for the annual percentage change [APC] slope) and decreased among children from 2010 through 2021 (*P* values for slopes: 2010–2017, *P* =* .*03; 2017–2021, *P *= .03). Prevalence of current asthma was higher among non-Hispanic Black people (children, 12.5%; adjusted prevalence ratio [APR] = 2.19; 95% CI, 1.68–2.84 and adults, 10.6%; APR = 1.25; 95% CI, 1.09–1.43) compared with non-Hispanic White people (children, 5.7%; adults, 8.2%). Prevalence of asthma attacks and use of asthma-related health care declined among adults and children. Asthma prevalence and asthma-related emergency department visits, hospitalization, and death rates differed by select characteristics.

**Conclusions:**

Although asthma attacks, ED visits, hospitalizations, and deaths have declined since 2010 among all ages, current asthma prevalence declined only among children, and significant disparities in health and health care use still exist.

**Summary**

**What is already known on this topic?**

Asthma is associated with substantial illness and death and disproportionately affects some populations more than others in the US.

**What is added by this report?**

Our article demonstrates ongoing disparities and trends in the current status of asthma, asthma-related health care use, and deaths.

**What are the implications for public health practice?**

Our findings provide information that may improve the delivery of care to reduce preventable asthma-related emergency department visits, hospitalizations, and deaths.

## Introduction

Asthma, a chronic respiratory disease, is associated with substantial illness and death (1–3), requiring ongoing medical management. The disorder is associated with a large economic cost (4) and a substantial number of missed school and workdays (5). The disorder is the focus of the Healthy People 2030 initiative to reduce asthma attacks, emergency department (ED) visits, hospitalizations, and deaths (6). Asthma disproportionately affects people from some racial and ethnic minority groups (3,7), people with low incomes (3,7), and people facing certain environmental factors (7,8).

Asthma is uncontrolled in approximately 50% of children (9) and 62% of adults (10) and results in frequent and intense episodes of symptoms (1), most commonly among children aged 0 to 4 years (59.1%) and Black people (62.9%) (9). Furthermore, ED visits, hospitalizations, and number of missed school days are higher among children with uncontrolled asthma (11). Children’s ED visits for asthma declined substantially from 2006 through 2018, but disparities in these children’s sociodemographic characteristics persist (3). Although asthma deaths have declined, these, too, are related to socioeconomic and demographic health disparities (3,7,12). Progress in asthma treatment has been slow, and asthma hospital admissions and deaths have declined only slightly in the past decade (13).

The Centers for Disease Control and Prevention’s (CDC’s) National Asthma Control Program was established in 1999 to fund asthma control in state, territorial, and municipal health departments. The program's goals are to reduce the number of deaths, hospitalizations, ED visits, school days or workdays missed, and limitations on activity due to asthma. This includes monitoring the health of people with asthma and determining health and health care disparities by analyzing data from multiple national and state-based surveys, hospital discharge records, and death vital statistics. Our objective was to describe asthma in the US by assessing prevalence of current asthma (defined as people who have ever been diagnosed with asthma by a health care professional and report still having asthma) and asthma attacks, asthma-related health care use, and asthma deaths among children and adults by sociodemographic characteristics and by trends across time.

## Methods

### Data sources

We used 3 data sources to calculate prevalence of current asthma and asthma attacks, asthma hospitalization rates, and asthma ED visit rates per 10,000 of the 2020 US census resident population, and to calculate the asthma death rate per million of the 2021 US census resident population: 1) the National Health Interview Survey (NHIS), 2010–2021 (14); 2) the Healthcare Cost and Utilization Project’s (HCUP’s) National Emergency Department Samples (NEDS) (15) and National Inpatient Sample (NIS), 2010–2020 (16); and 3) CDC Wonder (CDC Wide-Ranging Online Data for Epidemiologic Research), 2010–2021 (17). NHIS is an annual cross-sectional, in-person, household survey of noninstitutionalized US civilians that uses a geographically clustered sampling design (14). NEDS, a stratified probability sample of a set of hospital-owned EDs, is a large US all-payer database that gives national estimates of ED visits. Its data come from US hospital-owned EDs with data in the HCUP State Emergency Department Databases and the State Inpatient Databases (15). NIS data are acquired from 48 partners (47 states and the District of Columbia) and represent more than 97% of the US population. Its data include a sample of all discharges from US community hospitals, excluding rehabilitation and long-term acute care hospitals (16).

### Study population

We analyzed NHIS data to calculate prevalence per 10,000 of the US census resident population for current asthma and asthma attacks among people with current asthma. We also analyzed CDC mortality data offered online from the National Vital Statistics System for asthma death rates per million US census population for 2010 through 2021 and trends across all years for 3 groups: all ages, children (aged <18 y) (hereinafter, children), and adults (aged ≥18 y) (hereinafter, adults) (17). We applied the following select characteristics to all calculations: sex (male or female, as shown in the medical record), age (0–4 y, 5–17 y, 18–34 y, 35–64 y, or ≥65 y), race (White, Black, other), ethnicity (Hispanic, non-Hispanic), and US census region (Northeast, Midwest, South, or West). The “other race” group includes Asian or Pacific Islanders, American Indians or Alaska Natives, and people of any other race (17), another single race, or multiple races. Data from the other-race group were combined to obtain sufficient sample size for reliable estimates.

### Current asthma and asthma attacks

 People were classified as having current asthma if they responded yes to 2 questions: “Has a doctor or other health professional ever told you that you had asthma?” and “Do you still have asthma?” People with asthma were classified as having asthma attacks if they responded yes to 1 question: “During the past 12 months, have you had an episode of asthma or an asthma attack?” (14). Prevalence was calculated by our select characteristics.

### Asthma-related emergency department visits, inpatient hospital stays, and deaths

We used NEDS to calculate ED visits per 10,000 and defined a visit as one in a which asthma is the primary diagnosis according to an ICD (International Classification of Diseases)-10-CM diagnosis code of J45 (15,18). We used NIS to calculate asthma hospitalization rates per 10,000, defined as hospital in-patient short stays (<30 days) with asthma as the primary diagnosis according to ICD 10-CM code J45 (16,18). Data from the National Vital Statistics System, accessed through CDC WONDER, were used to generate asthma death rates per million where asthma was the underlying cause of death (ICD-10 codes J45 and J46) (17). Data from 2010 through 2021 were used to calculate trends in asthma mortality rates, and estimates in 2021 were calculated by our select characteristics. Because our study was a secondary analysis of publicly available, de-identified data, it did not require CDC institutional review board approval.

### Statistical analysis

We used SAS version 9.4 and SAS-callable SUDAAN 11 (Research Triangle Institute) to account for the complex sampling design of the survey data. Descriptive statistics such as stratification by our select characteristics were used to show asthma-related outcomes as they were observed in the population. We evaluated trends from 2010 to 2021 in prevalence of current asthma and having 1 or more asthma attacks during the past year among all ages, children, and adults with current asthma. In 2019, the NHIS questionnaire was redesigned (https://www.cdc.gov/nchs/nhis/2019_quest_redesign.htm), but changes did not affect our estimates. We also determined asthma indicators by our select characteristics and US census region for 2021. Sample weights were provided in the data sets for each year and were used to adjust for survey nonresponse, poststratification, and probability of selection (14) to get a more accurate representation of the study population. Participant response categories of don’t know, refused, not ascertained, and missing values were treated as missing. Wald χ^2^ tests were conducted to determine associations among demographic characteristics, US census regions, and study outcomes (ie, prevalence of current asthma and prevalence of asthma attacks among people with current asthma).

We estimated trends for 2010 through 2020 in use factor rates for asthma-related health care, including asthma ED visits and hospitalizations, per 10,000 of the US 2020 census resident population. Rates in 2020 were estimated by our select demographic characteristics. Discharge-level weights were applied from the database to produce unbiased national annual estimates from sample data (19,20). Cell sizes less than or equal to 10 using HCUP data sets were suppressed.

Trends in rates per million of asthma deaths were calculated for 2010 through 2021, and for 2021 alone, among all ages, children, and adults. Prevalence of current asthma and asthma attacks, rates of asthma health care use, and asthma death rates were also calculated. The difference between 2 population groups was assessed by using nondirectional 2-tailed *z* tests (at the α < 0.05 level). We used Joinpoint Regression software, version 5.0.2.0 (National Cancer Institute) to analyze trends by using log-linear regression models to determine significance trends. Joinpoint software calculates the fewest number of linear segments necessary to characterize a trend and the year(s) where 2 segments with different slopes meet. Associations between current asthma or asthma and select covariates were examined by using multivariable logistic regression models. The association between each health outcome (eg, current asthma or asthma attack) was assessed in separate models in which health outcome regressed over independent variables along with sex, age, and race and ethnicity. Adjusted prevalence ratios (APRs) were estimated by adjusting for sex, age, and race and ethnicity for all ages, children, and adults. Each of these 3 variables was only adjusted by the 2 other variables in the model (eg, age is adjusted by sex and race and ethnicity). Statistical tests used a significance level of *P *< .05, and 95% CIs were calculated for all estimates.

## Results

### Current asthma

**Figure 1:**
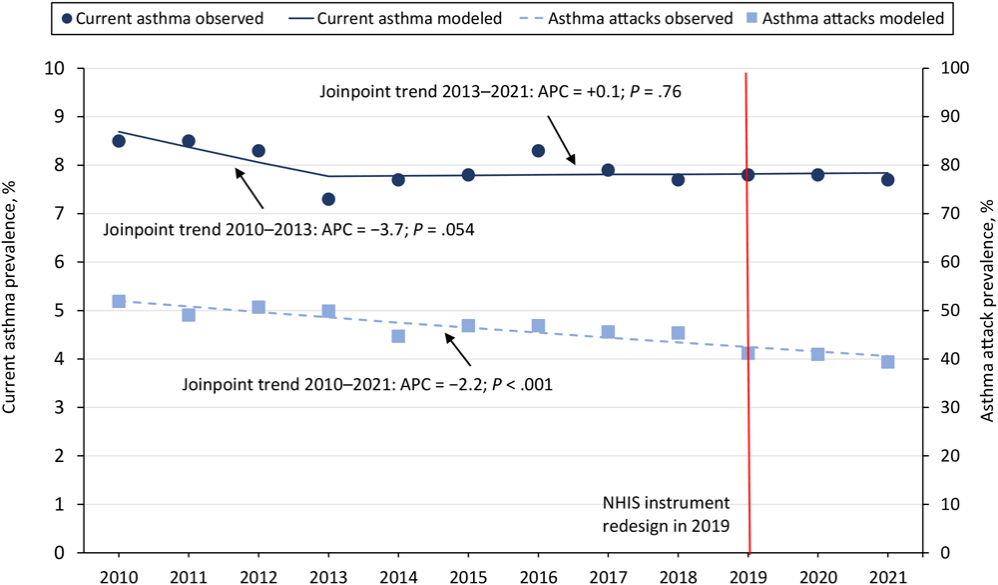
Prevalence of current asthma and asthma attacks among all ages by year. The *P* value of the trend line slope is significant at *P* < .05. The trend line is based on estimates from the statistical model and observed prevalence estimates (estimates as is from the survey data) (dots). The trend slope is numbered (slope 1, slope 2) when there is more than one significant trend line, as in the current asthma trend lines. Data source: National Center for Health Statistics, National Health Interview Survey, 2010–2021 (14).

In 2021, 24.9 million people in the US (4.7 million children and 20.3 million adults, 7.7% of the population) had asthma (Table 1). Asthma prevalence varied over time. Current asthma among all ages and among adults showed a nonsignificant decrease in 2010–2013 (Figure 1), then increased through 2021 significantly for adults (*P = .*04 for the slope). Among children, asthma prevalence significantly decreased from 2010 through 2021 (*P* =.03 for 2010–2017 trend slope, *P* =.03 for 2017–2021 trend slope).

**Table 1:** Prevalence of Current Asthma^a^ and Asthma Attacks^b^ Among All Ages, Children Aged 0–17 Years, and Adults Aged ≥18 Years, by Select Characteristics, National Health Interview Survey, 2021^c^

Characteristic	Current asthma				Asthma attacks			
	Estimated number^d^	% (SE)	APR (95% CI)^e^	*P* value	Estimated number^d^	% (SE)	APR (95% CI)^e^	*P* value
**All ages**								
Total	24,946,797	7.7 (0.18)	NA	NA	9,810,021	39.4 (1.05)	NA	NA
Sex								
Male	10,268,285	6.5 (0.22)	Reference	<.001^f^	3,507,730	34.2 (1.66)	Reference	<.001^f^
Female	14,678,512	8.9 (0.26)	1.36 (1.25–1.48)		6,302,291	43.0 (1.31)	1.27 (1.14–1.42)	
Age, y								
0–4	369,646	1.9 (0.34)	0.24 (0.17–0.34)	<.001^f^	233,364	63.1 (8.09)	1.73 (1.34–2.24)	<.001^f^
5–17	4,305,830	8.1 (0.42)	0.99 (0.85–1.14)		1,577,699	36.6 (2.55)	1.03 (0.86–1.24)	
18–34	6,157,181	8.4 (0.44)	Reference		2,269,833	36.9 (2.50)	Reference	
35–64	10,079,876	8.2 (0.27)	0.97 (0.86–1.10)		4,515,241	44.9 (1.62)	1.17 (1.01–1.37)	
≥65	4,034,264	7.2 (0.33)	0.83 (0.73–0.96)		1,213,884	30.3 (2.21)	0.77 (0.62–0.94)	
Race or ethnicity								
Hispanic	3,496,174	5.7 (0.35)	0.74 (0.65–0.84)	<.001^f^	1,370,503	39.2 (2.80)	0.96 (0.82–1.12)	.13
Non-Hispanic Black	4,234,040	11.1 (0.64)	1.41 (1.24–1.60)		1,438,527	34.0 (2.63)	0.82 (0.70–0.97)	
Non-Hispanic Other^g^	2,087,484	7.1 (0.53)	0.91 (0.78–1.07)		901,769	43.3 (3.66)	1.07 (0.89–1.27)	
Non-Hispanic White	15,129,098	7.8 (0.22)	Reference		6,099,221	40.4 (1.34)	Reference	
US census region								
Northeast	4,096,784	7.4 (0.47)	0.87 (0.75–1.02)	.08	1,490,033	36.3 (2.52)	0.90 (0.77–1.07)	.43
Midwest	5,744,256	8.5 (0.38)	0.99 (0.88–1.12)		2,188,525	38.2 (2.19)	0.94 (0.81–1.09)	
South	9,047,848	7.3 (0.30)	0.83 (0.74–0.93)		3,686,723	40.8 (1.84)	1.04 (0.91–1.20)	
West	6,057,909	7.8 (0.30)	Reference		2,453,177	40.6 (1.84)	Reference	
**Adults ≥18 years**								
Total	20,271,321	8.0 (0.34)	NA	NA	7,998,958	39.6 (2.02)	NA	NA
Sex								
Male	7,573,139	6.2 (0.25)	Reference	<.001^f^	2,473,035	32.8 (1.94)	Reference	<.001^f^
Female	12,698,183	9.8 (0.30)	1.57 (1.43–1.73)		5,525,924	43.6 (1.40)	1.35 (1.18–1.53)	
Race and ethnicity								
Hispanic	2,486,966	5.8 (0.43)	0.68 (0.59–0.80)	<.001^f^	987,746	39.7 (3.38)	0.95 (0.79–1.14)	.17
Non-Hispanic Black	3,130,895	10.6 (0.69)	1.25 (1.09–1.43)		1,039,101	33.3 (3.06)	0.80 (0.66–0.97)	
Non-Hispanic Other^g^	1,638,065	7.6 (0.67)	0.90 (0.75–1.08)		705,552	43.2 (4.21)	1.07 (0.87–1.30)	
Non-Hispanic White	13,015,396	8.2 (0.24)	Reference		5,266,559	40.6 (1.45)	Reference	
US census region								
Northeast	3,534,987	8.0 (0.52)	0.89 (0.76–1.05)	.08	1,288,580	36.5 (2.86)	0.89 (0.75–1.08)	.32
Midwest	4,633,048	8.8 (0.43)	0.97 (0.85–1.11)		1,719,494	37.2 (2.39)	0.90 (0.76–1.06)	
South	7,123,176	7.4 (0.34)	0.80 (0.71–0.92)		2,915,835	41.0 (2.11)	1.02 (0.88–1.19)	
West	4,980,112	8.3 (0.39)	Reference		2,075,049	41.8 (2.27)	Reference	
**Children <18 y**								
Total	4,675,475	6.5 (0.32)	NA	NA	1,811,063	38.7 (2.51)	NA	NA
Sex								
Male	2,695,146	7.3 (0.49)	Reference	.007^f^	1,034,696	38.4 (3.40)	Reference	.87
Female	1,980,329	5.6 (0.41)	0.77 (0.64–0.93)		776,367	39.2 (3.70)	1.02 (0.79–1.32)	
Race or ethnicity								
Hispanic	1,009,208	5.4 (0.55)	0.95 (0.75–1.21)	.001	382,757	37.9 (5.61)	0.95 (0.68–1.34)	.88
Non-Hispanic Black	1,103,145	12.5 (1.36)	2.19 (1.68–2.84)		399,426	36.2 (5.85)	0.90 (0.62–1.30)	
Non-Hispanic Other^g^	449,419	5.7 (0.81)	1.01 (0.75–1.37)		196,217	43.7 (7.56)	1.05 (0.70–1.56)	
Non-Hispanic White	2,113,703	5.7 (0.42)	Reference		832,662	39.4 (3.64)	Reference	
US census region								
Northeast	561,797	5.0 (0.75)	0.76 (0.54–1.07)	.11	195,906	34.9 (6.57)	0.95 (0.61–1.48)	.69
Midwest	1,111,208	7.4 (0.69)	1.11 (0.85–1.44)		469,031	42.2 (5.31)	1.14 (0.80–1.62)	
South	1,924,672	6.8 (0.56)	0.91 (0.71–1.16)		767,998	39.9 (4.16)	1.14 (0.81–1.60)	
West	1,077,798	6.2 (0.59)	Reference		378,128	35.1 (4.42)	Reference	

Abbreviation: APR: adjusted prevalence ratio; NA: not applicable; SE: standard error.

a People who responded yes to the following questions: “Has a doctor or other health professional ever told you that you had asthma?” and “Do you still have asthma?”

b Among those with current asthma, respondents were classified as having asthma attacks if they responded yes to “During the past 12 months, have you had an episode of asthma or an asthma attack?”

c National Center for Health Statistics (14).

d May not sum to total because of rounding and missing values.

e Adjusted for age, sex, and race or ethnicity when regressing the dependent variables (ie, current asthma and asthma attacks) over each independent variable by using multivariable logistic regression models. For age, sex, and race or ethnicity variables, only adjusted for the other 2.

f Significant at *P* <.05. Calculated by using the Wald χ^2^ test of the association between outcomes and characteristics.

g Non-Hispanic Asian, Non-Hispanic American Indian or Alaska Native, any other race or ethnicity, other single race, or multiple races.

**Table:** 

Year	Current asthma		Asthma attacks	
	Observed	Modeled	Observed	Modeled
2010	51.9	51.96	8.5	8.69
2011	49.1	50.82	8.5	8.37
2012	50.7	49.69	8.3	8.06
2013	49.9	48.59	7.3	7.77
2014	44.7	47.51	7.7	7.78
2015	46.9	46.46	7.8	7.79
2016	46.9	45.43	8.3	7.8
2017	45.6	44.42	7.9	7.81
2018	45.4	43.43	7.7	7.81
2019	41.2	42.47	7.8	7.82
2020	41.0	41.53	7.8	7.83

Current asthma prevalence varied by our selected demographic characteristics. Prevalence was significantly associated with sex (adults, *P* <.001; children, *P* = .007); age (*P* < .001 all ages), and race and ethnicity (adults, *P* < .001; children, *P* < .001). Among adults, current asthma prevalence was higher among females than males (9.8% females vs 6.2% males; APR = 1.57 [95% CI, 1.43–1.73]) and among non-Hispanic Black adults than non-Hispanic White adults (10.6% vs 8.2%; APR = 1.25 [95% CI, 1.09–1.43]) (Table 1). Current asthma prevalence was lower among Hispanic adults compared with non-Hispanic White adults (5.8% vs 8.2%; APR = 0.68 [95% CI, 0.59–0.80]) and adults who lived in the South compared with adults who lived in the West (7.4% vs 8.3%; APR = 0.80 [95% CI, 0.71–0.92]). Prevalence was significantly lower among children aged 0 to 4 years (1.9%) and among adults aged 65 years or older (7.2%) than among adults aged 18 to 34 years (8.4%) (Table 1).

Among children, current asthma prevalence was higher among non-Hispanic Black children (12.5%; APR = 2.19 [95% CI, 1.68–2.84]) compared with non-Hispanic White children (5.7%) (Table 1). Current asthma prevalence was lower among female children (5.6%; APR = 0.77 [95% CI, 0.64–0.93]) compared with male children (7.3%).

### Asthma attacks

In 2021, about 39.4% of people with current asthma reported having 1 or more asthma attacks in the past 12 months (39.6% among adults and 38.7% among children) (Table 1). Prevalence of asthma attacks significantly decreased over time. Among people of all ages with asthma, the prevalence of attacks decreased significantly, from 51.9% in 2010 to 39.4% in 2021 (*P* < .001 for the trend slope) (Figure 1). Among adults with asthma, attack prevalence decreased from 58.3% in 2010 to 38.7% in 2021 (*P* < .001 for the trend slope); among children, attack prevalence decreased from 49.1% in 2010 to 39.6% in 2021 (*P* = .003 for the trend slope).

Prevalence of asthma attacks among adults with current asthma was significantly associated with sex (*P* < .001) and age (*P* < .001). Asthma attack prevalence was significantly higher among female adults (43.6%; APR = 1.35 [95% CI, 1.18–1.53]) compared with male adults (32.8%) and was significantly lower among non-Hispanic Black adults (33.3%; APR = 0.80 [95% CI, 0.66–0.97]) than non-Hispanic White adults (40.6%). Asthma attack prevalence was significantly higher among children aged 0 to 4 years (63.1%; APR = 1.73 [95% CI, 1.34–2.24]) and among adults aged 35 to 64 years (44.9%; APR = 1.17 [95% CI, 1.01–1.37]), and significantly lower among adults aged 65 years or older (30.3%; APR = 0.77 [95% CI, 0.62–0.94]) compared with adults aged 18 to 34 years (36.9%) (Table 1). We found no significant differences in prevalence of asthma attacks by census region among all ages, children, or adults.

### Asthma-related emergency department visits

**Figure 2:**
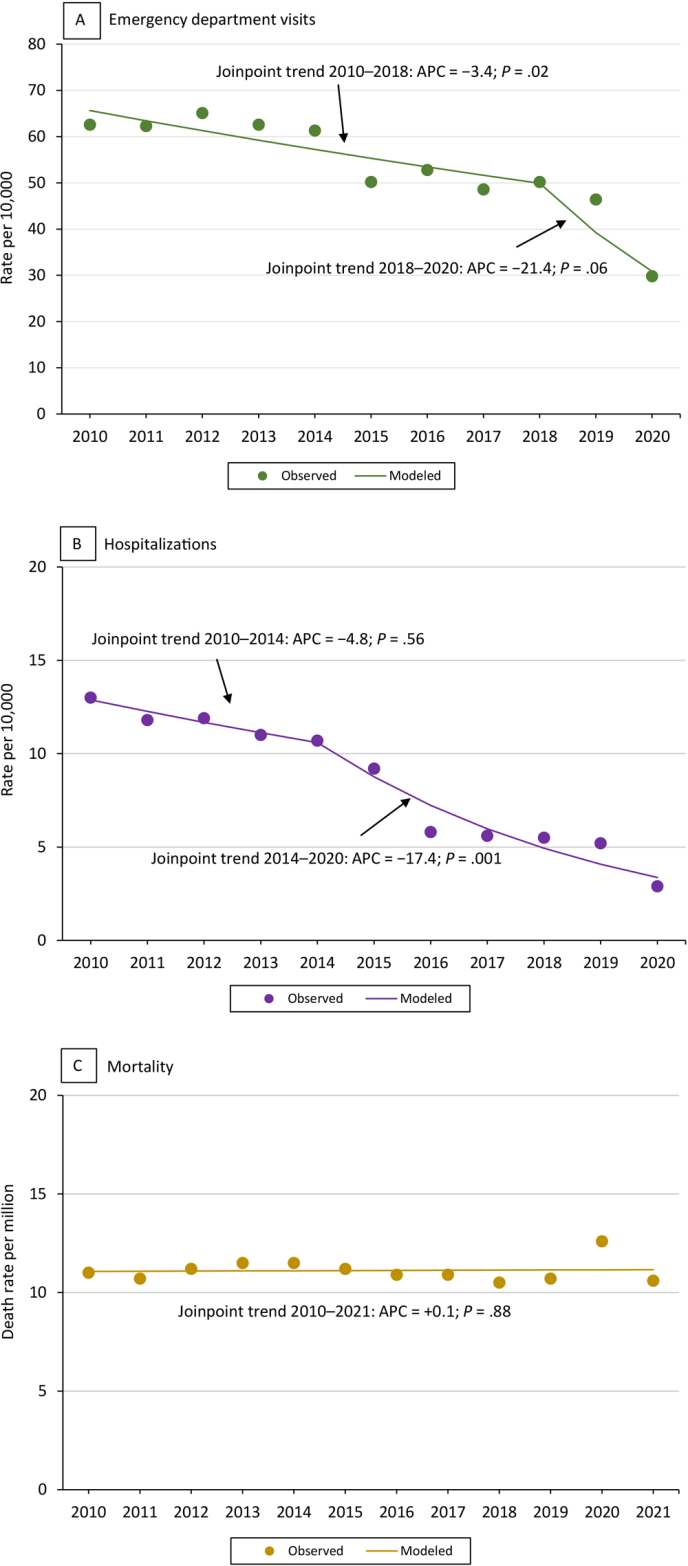
Asthma-related health care use and death rate among all ages by year. The *P* value of trend line slope is significant at .05. The trend line is based on estimates from the statistical model and observed prevalence estimates (estimates as is from the survey data) (dots). The trend slopes are numbered (slope 1, slope 2) when there is more than 1 significant trend line, as in the current asthma trend lines. The health care use rate is shown as the number of hospitalizations and emergency department visits per the US Census resident population for the given year. Data sources: asthma emergency department visits and hospitalizations: Healthcare Cost and Utilization Project, National (Nationwide) Inpatient Sample (16) and National (Nationwide) Emergency Department Sample (15), Agency for Healthcare Research and Quality. Asthma deaths: CDC Wonder (Wide-Ranging Online Data for Epidemiologic Research) (17).

Approximately 1 million people in the US had an ED visit for asthma in 2020 (29.8 per 10,000 US census 2020 resident population) (Table 2). The asthma ED visit rate per 10,000 for all ages decreased significantly in 2018, from 62.7 in 2010 to 50.2 in 2018 (*P *= .02 for the slope), but no significant changes occurred between 2018 and 2020 (*P* = .06 for the slope) (Figure 2). Among adults, the asthma ED visit rate per 10,000 decreased significantly, from 52.3 in 2010 to 27.8 in 2020 (*P* <.001 for the slope). The asthma ED visit rate per 10,000 among children declined from 2010 through 2020 but was only significant between 2018 and 2020 (*P* = .03 for the slope).

**Table 2:** Asthma Emergency Department Visits and Hospitalization Rates^a^ by Patient Characteristics Among All Ages, Children Aged 0–17 Years, and Adults Aged ≥18 Years, US, 2020

Characteristic	Emergency department visits			Hospitalizations		
	Estimated number^b^	Rate per 10,000 (SE)^c^	*P* value for *z* score	Estimated number^b^	Rate per 10,000 (SE)^c^	*P* value for *z* score
**All ages**						
Total	986,453	29.8 (0.98)	NA	94,560	2.9 (0.06)	NA
Sex^d^						
Male^e^	445,261	27.1 (1.01)	Reference	36,110	2.2 (0.06)	Reference
Female	541,130	32.3 (1.00)	<.001^f^	58,450	3.5 (0.07)	<.001^f^
Age, y						
0–4	80,898	42.0 (3.66)	<.001^f^	11,020	5.7 (0.34)	<.001^f^
5–17	189,432	34.5 (2.39)	<.001^f^	16,035	2.9 (0.18)	<.001^f^
18–34^e^	299,453	39.5 (1.27)	Reference	14,445	1.9 (0.05)	Reference
35–64	348,767	27.4 (0.94)	<.001^f^	37,630	3.0 (0.06)	<.001^f^
≥65	67,896	12.5 (0.44)	<.001^f^	15,430	2.8 (0.07)	<.001^f^
Race, excluding ethnicity						
Black	371,608	82.8 (4.89)	<.001^f^	31,725	7.1 (0.24)	<.001^f^
Other^g^	246,004	70.5 (4.25)	<.001^f^	22,970	6.6 (0.27)	<.001^f^
White^e^	348,760	13.9 (0.40)	Reference	37,365	1.5 (0.03)	Reference
Ethnicity						
Hispanic	179,553	29.0 (2.12)	<.001^f^	15,890	2.6 (0.12)	<.001^f^
Non-Hispanic	786,819	29.2 (1.00)	<.001^f^	76,170	2.8 (0.06)	<.001^f^
**Adults ≥18 y**						
Total	716,117	27.8 (0.89)	NA	67,505	2.6 (0.05)	NA
Sex^d^						
Male^e^	281,893	22.3 (0.80)	Reference	20,045	1.6 (0.04)	Reference
Female	434,185	33.1 (1.03)	<.001^f^	47,460	3.6 (0.07)	<.001^f^
Race, excluding ethnicity						
Black	270,279	80.6 (4.96)	<.001^f^	21,940	6.5 (0.20)	<.001^f^
Other^g^	159,930	64.3 (3.44)	<.001^f^	14,970	6.0 (0.24)	<.001^f^
White^e^	272,380	13.7 (0.38)	Reference	29,425	1.5 (0.03)	Reference
Ethnicity						
Hispanic	112,731	26.3 (1.67)	<.001^f^	10,465	2.4 (0.12)	<.001^f^
Non-Hispanic	589,858	27.5 (0.95)	<.001^f^	55,870	2.6 (0.04)	<.001^f^
**Children <18 y**						
Total	270,330	36.4 (2.69)	NA	27,055	3.6 (0.21)	NA
Sex^d^						
Male^e^	163,367	43.0 (3.23)	Reference	16,065	4.2 (0.25)	Reference
Female	106,938	29.5 (2.14)	<.001^f^	10,990	3.0 (0.18)	<.001^f^
Race, excluding ethnicity						
Black	101,329	89.5 (8.09)	<.001^f^	9,785	8.6 (0.65)	<.001^f^
Other^g^	86,074	86.0 (9.75)	<.001^f^	8,000	8.0 (0.60)	<.001^f^
White^e^	76,374	14.4 (1.10)	Reference	7,940	1.5 (0.09)	Reference
Ethnicity						
Hispanic	66,821	35.2 (4.62)	<.001^f^	5,425	2.9 (0.24)	<.001^f^
Non-Hispanic	196,955	35.7 (2.52)	<.001^f^	20,300	3.7 (0.22)	<.001^f^

Abbreviation: NA, not applicable; SE: standard error.

a Asthma as the primary diagnosis (ICD-10-CM Code: J45) (15,18); Nationwide Emergency Department Sample, Healthcare Cost and Utilization Project, 2010–2020 (15); and Nationwide Inpatient Sample, Healthcare Cost and Utilization Project, 2010–2020 (16).

b Used sample weights provided in the data set to estimate numbers of respondents within select characteristics; may not sum to total because of rounding and missing values.

c Crude rate per 10,000 US census 2020 resident population.

d Sex categories are male and female as designated in the medical record.

e Reference category.

f Significant at *P *< .05. *P* values calculated by using *z* test.

g Includes Asian or Pacific Islander, American Indian or Alaska Native, and other races.

**Table:** 

**Year**	**Observed**	**Modeled**
Asthma emergency department visits		
2010	62.6	65.64
2011	62.3	63.42
2012	65.1	61.29
2013	62.6	59.22
2014	61.3	57.22
2015	50.2	55.29
2016	52.8	53.43
2017	48.6	51.63
2018	50.2	49.89
2019	46.4	39.22
2020	29.8	30.83
Asthma inpatient hospital stays		
2010	13	12.88
2011	11.8	12.27
2012	11.9	11.68
2013	11	11.13
2014	10.7	10.6
2015	9.2	8.76
2016	5.8	7.23
2017	5.6	5.98
2018	5.5	4.94
2019	5.2	4.08
2020	2.9	3.37
Asthma mortality		
2010	11	11.07
2011	10.7	11.08
2012	11.2	11.09
2013	11.5	11.1
2014	11.5	11.1
2015	11.2	11.11
2016	10.9	11.12
2017	10.9	11.13
2018	10.5	11.14
2019	10.7	11.15
2020	12.6	11.15
2021	10.6	11.16

The asthma ED visit rate per 10,000 varied by demographic characteristics (Table 2). It was significantly lower among adults aged 35 or older (35–64 y, 27.4; ≥65 y, 12.5) compared with adults aged 18 to 34 years (39.5). Among adults aged 18 or older, the asthma rate was higher for females (33.1) than males (22.3). The rate was also higher among Black (80.6), other races (64.3), and Hispanic (26.3) adults compared with White adults (13.7) (Table 2).

Among children, the asthma ED visit rate per 10,000 was significantly higher for males (43.0) than for females (29.5) and for Black (89.5), other race (86.0), and Hispanic (35.2) children than for White children (14.4).

### Asthma hospital inpatient stays

Nearly 100,000 people in the US were hospitalized for asthma in 2020 (2.9 per 10,000 US census 2020 resident population) (Table 2). The rate was 13.0 per 10,000 for all ages in 2010 and 10.7 per 10,000 in 2014, but no significant changes occurred between 2010 and 2014 (*P* = .56 for the slope). The rate then decreased significantly, to 2.9 per 10,000 in 2020 (*P *= .001 for the slope) (Figure 2).

The trend in asthma hospitalizations for children and adults also declined across time. Among adults, the asthma hospitalization rate per 10,000 decreased significantly, from 12.0 in 2010 to 2.6 in 2020 (*P* < .001 for the slope). The rate among children decreased significantly from 2010 through 2018 and further decreased through 2020 (*P* = .006, 2010–2018; *P* = .005, 2018–2020 for the slopes).

The asthma hospitalization rate varied by demographic characteristics (Table 2). Asthma hospitalization rates per 10,000 among all other age groups (0–4 years: 5.7; 5–17: 2.9; 35–64: 3.0; ≥65 years: 2.8) were significantly higher than among people aged 18 to 34 years (1.9) (Table 2). Among adults, the asthma hospitalization rate was significantly higher among females (3.6) than males (1.6). The rate was also higher among Black (6.5), other race (6.0), and Hispanic (2.4) adults compared with White adults (1.5) (Table 2).

Among children, the asthma hospitalization rate per 10,000 was significantly higher for males (4.2) than for females (3.0). The rate was also significantly higher for Black (8.6), other race (8.0), and Hispanic (2.9) children than for White (1.5) children.

### Asthma as the underlying cause of death

In 2021, asthma was the underlying cause of death for 3,517 (10.6 per million) people in the US (Table 3). The trend in asthma death rates among all ages (*P* =.88 for the slope), among adults (*P* = .99 for the slope), and among children (*P* = .35 for the slope) were stable from 2010 through 2021 (Figure 2).

**Table 3:** Asthma Deaths by Select Characteristics Among All Ages, Children Aged 0–17 Years, and Adults Aged ≥18 Years — US, 2021^a^

Characteristic^b^	Number of deaths^c^	Death rate per million (SE)^c^^,^ d	*P* value for *z *score
**All ages**			
Total	3,517	10.6 (0.18)	NA
Sex^e^			
Male^g^	1,430	8.7 (0.23)	Reference
Female	2,087	12.5 (0.27)	<.001^f^
Age, y			
0–4	26	1.4 (0.27)	<.001^f^
5–17	119	2.2 (0.20)	<.001^f^
18–34^g^	406	5.4 (0.27)	Reference
35–64	1,453	11.5 (0.30)	<.001^f^
≥65	1,513	27.1 (0.70)	<.001^f^
Race and ethnicity			
Hispanic^i^	366	5.8 (0.31)	<.001^f^
Non-Hispanic Black	1,020	24.4 (0.76)	<.001
Non-Hispanic Other^h^	194	6.3 (0.46)	<.001
Non-Hispanic White^g^	1,929	9.8 (0.22)	Reference
**Adults ≥18 y**			
Total	3,372	13.1 (0.22)	NA
Sex^e^			
Male^g^	1,341	10.6 (0.29)	Reference
Female	2,031	15.4 (0.34)	<.001^f^
Race and ethnicity			
Hispanic^i^	339	7.8 (0.42)	<.001^f^
Non-Hispanic Black	942	29.7 (0.97)	<.001^f^
Non-Hispanic Other^h^	190	8.5 (0.62)	<.001^f^
Non-Hispanic White^g^	1,893	11.8 (0.27)	Reference
**Children <18 y**			
Total	145	2.0 (0.16)	NA
Sex^e^			
Male^g^	89	2.4 (0.25)	Reference
Female	56	1.6 (0.21)	0.01^f^
Race and ethnicity			
Hispanic^i^	27	1.4 (0.27)	.21
Non-Hispanic Black	78	7.7 (0.87)	<.001
Non-Hispanic Other^h^	— j	— j	NA
Non-Hispanic White^g^	36	1.0 (0.17)	Reference

Abbreviations: NA: not applicable; SE: standard error.

a Centers for Disease Control and Prevention. CDC Wonder (https://wonder.cdc.gov/).

b Numbers in select characteristics may not sum to total because of rounding and missing values.

c Asthma as the underlying cause of death (ICD-10 codes J45–J46). (https://wonder.cdc.gov/wonder/help/ucd-expanded.html#).

d Crude death rate per million, US census 2021 resident population.

e Sex categories are male and female as reported in death certificate.

f Significant at *P *< .05. *P* values calculated by using *z* test.

g Reference category.

h Non-Hispanic Asian, non-Hispanic American Indian/Alaska Native, non-Hispanic native Hawaiian/Pacific Islander, and non-Hispanic people of more than 1 race.

i Information for Hispanic origin was missing for suppressed number of deaths.

j Suppressed because the number of deaths was 9 or fewer.

The asthma death rate per million varied by demographic characteristics (Table 3). The rate was significantly lower among children (0–4 y, 1.4; 5–17 y, 2.2), and significantly higher among adults aged 35 years or older (35–64 y, 11.5; ≥65 y, 27.1) compared with people aged 18 to 34 years (5.4). Rates for adults aged 18 years or older were higher for females (15.4) than males (10.6). The rate was also higher among non-Hispanic Black adults (29.7), and lower for adults of non-Hispanic other race (8.5) and Hispanic adults (7.8) compared with non-Hispanic White adults (11.8) (Table 3).

Among children, the asthma death rate per million was significantly higher for males (2.4) than for females (1.6) and also was significantly higher for non-Hispanic Black children (7.7) than for non-Hispanic White children (1.0).

## Discussion

We found that current asthma prevalence among adults increased significantly from 2013 through 2021 and decreased significantly among children from 2010 through 2021. Akinbami et al (21) found an increased trend in asthma prevalence among children from 2001 through 2008, plateauing thereafter with a possible decline starting in 2013. Improvements in asthma diagnostic testing (1,2) or changes in exposure to environmental factors linked to developing asthma might explain the trends reported in that study (22).

Another study found that prevalence of asthma attacks did not show a significant trend in either direction among adults or children in the first decade of this century (23). However, another study found a decrease in prevalence of asthma attacks among children with current asthma from 2001 through 2016 (24). We found that prevalence of asthma attacks continued to decline, showing a significant decrease in prevalence from 2010 through 2021 among both children and adults. Asthma-related ED visits and hospitalizations among children and adults decreased significantly, but the decrease in ED visits among children was only significant in later years, 2018 through 2020. Although the COVID-19 pandemic could account for asthma-related ED visits and hospitalizations in 2020, other possible reasons for the declines over the years may include improved, ongoing public health programs and use of emerging evidence-based strategies in asthma diagnosis, management, and treatment (2). Studies have also found that ED visits for asthma were lower during the COVID-19 pandemic compared with pre-pandemic years (before 2020), especially among children. This was explained by changes in the health care–seeking behaviors possibly due to the pandemic, such as exposure avoidance and fears of visiting an ED. Also, ED visits for asthma attacks triggered by COVID-19 may have been classified as COVID-19 (25). A separate trend analysis of asthma-related ED visits and hospitalization data showed significant declines among children and adults from 2010 through 2019.

Our study also found that in 2021, current asthma prevalence was lower but asthma attack prevalence was higher among children aged 0 to 4 years compared with adults aged 18 to 34 years. Past studies also found similar patterns in such children (3). Lower asthma prevalence among children aged 0 to 4 years could be because asthma in children in this age group is often underdiagnosed; its symptoms, such as wheezing and coughing, can be caused by other respiratory tract infections (eg, rhinitis, croup, pneumonia, bronchiolitis) and because of difficulties in using diagnostic lung tests (eg, spirometry) accurately in children in this age group (1). Current asthma prevalence was lower among adults aged 65 years or older than those aged 18 to 34 years. Asthma in older adults may be underdiagnosed because of underuse of diagnostic tests, challenges in application and interpretation of tests, and difficulty distinguishing asthma symptoms from similar symptoms because of other conditions (eg, congestive heart failure, emphysema, chronic bronchitis, chronic aspiration, gastroesophageal reflux disease, tracheobronchial tumors) (26,27).

Among adults, prevalence of current asthma and asthma attacks was higher for females compared with males after adjusting for age and race and ethnicity but lower for children. Asthma attacks also were not associated with sex. The reason for this sex pattern in asthma could be the effect of sex-specific hormones and pathophysiology (28). 

Current asthma prevalence was higher among non-Hispanic Black children and adults and lower among Hispanic adults, after adjusting for sex and age, compared with non-Hispanic White adults. Although asthma attack prevalence did not differ by race and ethnicity, after adjusting for age and sex, prevalence was lower among non-Hispanic Black adults compared with non-Hispanic White adults. Disparities persist since our last asthma surveillance summary, which also demonstrated that prevalence remains higher among non-Hispanic Black children and adults and lower among Hispanic adults than non-Hispanic White adults (3). People with low incomes and from some racial and ethnic minority groups disproportionately experience adverse health outcomes, which may be associated with factors such as poor housing quality (7,29,30), adverse environmental exposures (7,8,12,29,30), reduced access to health care (7,31), and health care quality (7,30,31). We did not examine factors contributing to lower asthma attacks among non-Hispanic Black people. Further research is needed to identify those factors.

Further disparities were found in health care use. Children had higher rates of asthma ED visits and hospitalizations than adults, which was consistent with the findings of McDermott et al (32) and Qin et al (33). McDermott et al (32) determined that asthma was the most common reason for potentially preventable pediatric hospitalization in 2017. Despite recent advancements in medical care, an estimated 94,560 people were hospitalized and 986,453 people had ED visits for asthma in 2020, which represents a substantial burden on patients and the health care system. We also found that people of races and ethnicities other than non-Hispanic White had significantly higher rates of asthma ED visits and hospitalizations than White people. Qin et al (33) found that non-Hispanic Black and Hispanic people were more likely to have asthma-related ED visits (33). Asthma disorders and health care use are affected by socioeconomic and demographic factors that contribute to health disparities (7,31). Among children with asthma, more non-Hispanic White children used the doctor’s office as their usual place for medical care than non-Hispanic Black and Hispanic children (34). Also, more Black children with insurance had cost barriers to seeing a doctor compared with White children (35).

As other studies show, the contributing factors to health disparities in asthma may include smoking (29,30), economic instability (7), poor housing and environmental conditions (7,8,29,30), health care access (7,12,31), quality of care (7,12,30), and medication adherence (30). Guidelines-based care, including evidence-based interventions and tailored asthma management, may alleviate the burden of asthma and reduce disparities (2).

We found that the asthma death rate was higher for female than male adults but higher among male than female children. We also found this sex pattern for current asthma and health care use. The asthma death rate was also higher among older adults, which was observed in other studies that showed asthma mortality increased with age (26). Baptist and Busse noted that management and medical care for asthma among older adults might be difficult and complicated because of comorbidities and age-related pathophysiologic changes, which can increase risk of death from asthma (26). Although some declines in asthma outcomes have been observed, that article showed continued opportunities to address health disparities.

Our study’s strengths are that asthma prevalence and attack data come from NHIS, which is the major and longest-running household health survey in the US (14), and from its decades worth of data to determine trends. These data include multiple major indicators (current asthma, asthma attacks, asthma-related health care use, and asthma mortality), hospital administrative data, and vital statistics data.

Our study had limitations. NHIS responses are self-reported; therefore, some misclassification and associated biases may result. Because NHIS is a cross-sectional study, a cause-and-effect relationship between outcome and independent variables cannot be inferred. In addition, asthma estimates for children are based on proxy (adult) responses and may be misclassified, although the proxy adult selected was the one most knowledgeable about the child's health. Trend results need to be interpreted considering the transition in diagnostic coding from ICD-9-CM to ICD-10-CM in October 2015 and redesign of NHIS in 2019. Additionally, the analysis time period of our study includes the COVID-19 pandemic, which began in 2020 and likely affected survey processes and asthma ED visits.

### Conclusion

Although asthma attacks, asthma ED visits, asthma hospitalizations, and asthma deaths declined since 2010 among all ages, current asthma prevalence declined only among children. Significant disparities in health and health care use still exist. Our study’s results can help decision makers and public health practitioners provide tailored interventions and health care initiatives to improve the health of people with asthma and reduce preventable health care use.
